# Hydrogen Limitation and Syntrophic Growth among Natural Assemblages of Thermophilic Methanogens at Deep-sea Hydrothermal Vents

**DOI:** 10.3389/fmicb.2016.01240

**Published:** 2016-08-05

**Authors:** Begüm D. Topçuoğlu, Lucy C. Stewart, Hilary G. Morrison, David A. Butterfield, Julie A. Huber, James F. Holden

**Affiliations:** ^1^Department of Microbiology, University of Massachusetts, AmherstMA, USA; ^2^Marine Biological Laboratory, Josephine Bay Paul Center, Woods HoleMA, USA; ^3^Joint Institute for the Study of Atmosphere and Ocean, University of Washington, SeattleWA, USA; ^4^Pacific Marine Environmental Laboratory, National Oceanic and Atmospheric Administration, SeattleWA, USA

**Keywords:** hydrogen, syntrophy, methanogenesis, hydrothermal vents, *Methanococcales*, *Thermococcales*

## Abstract

Thermophilic methanogens are common autotrophs at hydrothermal vents, but their growth constraints and dependence on H_2_ syntrophy *in situ* are poorly understood. Between 2012 and 2015, methanogens and H_2_-producing heterotrophs were detected by growth at 80°C and 55°C at most diffuse (7–40°C) hydrothermal vent sites at Axial Seamount. Microcosm incubations of diffuse hydrothermal fluids at 80°C and 55°C demonstrated that growth of thermophilic and hyperthermophilic methanogens is primarily limited by H_2_ availability. Amendment of microcosms with NH_4_^+^ generally had no effect on CH_4_ production. However, annual variations in abundance and CH_4_ production were observed in relation to the eruption cycle of the seamount. Microcosm incubations of hydrothermal fluids at 80°C and 55°C supplemented with tryptone and no added H_2_ showed CH_4_ production indicating the capacity *in situ* for methanogenic H_2_ syntrophy. 16S rRNA genes were found in 80°C microcosms from H_2_-producing archaea and H_2_-consuming methanogens, but not for any bacteria. In 55°C microcosms, sequences were found from H_2_-producing bacteria and H_2_-consuming methanogens and sulfate-reducing bacteria. A co-culture of representative organisms showed that *Thermococcus paralvinellae* supported the syntrophic growth of *Methanocaldococcus bathoardescens* at 82°C and *Methanothermococcus* sp. strain BW11 at 60°C. The results demonstrate that modeling of subseafloor methanogenesis should focus primarily on H_2_ availability and temperature, and that thermophilic H_2_ syntrophy can support methanogenesis within natural microbial assemblages and may be an important energy source for thermophilic autotrophs in marine geothermal environments.

## Introduction

Approximately 1 Gt of CH_4_ is formed globally per year from H_2_, CO_2_ and acetate through methanogenesis, largely by methanogens growing in syntrophic association with anaerobic microbes that hydrolyze and ferment biopolymers (Thauer et al., 2008). At deep-sea hydrothermal vents, methanogens are continuously flushed from the ocean crust where H_2_ concentrations in hydrothermal fluids are high, but are scarce in low H_2_ environments, as measured by culture-dependent techniques (Stewart et al., 2016), culture-independent techniques (Perner et al., 2007; Flores et al., 2011), and both techniques in tandem (Takai et al., 2004, 2008, 2009; Nakagawa et al., 2005, 2006; Ver Eecke et al., 2012; Lin et al., 2016). Thermophilic methanogens are consistently found in hydrothermal fluids at Axial Seamount, an active deep-sea volcano in the northeastern Pacific Ocean, and nearly all belong to the genera *Methanocaldococcus, Methanothermococcus*, and *Methanococcus* (Huber et al., 2002; Ver Eecke et al., 2012; Meyer et al., 2013; Fortunato and Huber, 2016). Axial Seamount erupted in 1998, 2011, and 2015 (Chadwick et al., 2012, 2013; Kelley et al., 2015), and basalt formed by these eruptions hosted hydrothermal niches that support methanogenesis (Huber et al., 2002; Meyer et al., 2013).

Hyperthermophilic heterotrophs capable of H_2_ production, mostly *Thermococcales*, are generally co-localized with thermophilic and hyperthermophilic methanogens in low-temperature hydrothermal vent fluids (Takai et al., 2004, 2008, 2009; Nakagawa et al., 2005, 2006; Flores et al., 2011; Ver Eecke et al., 2012; Lin et al., 2016). Some *Thermococcus* species produce H_2_ and possess up to five different hydrogenases (Lee et al., 2008; Kim et al., 2010, 2013; Hensley et al., 2014, 2016) and may serve as an alternative source of H_2_ for methanogens in low H_2_ environments. Laboratory studies demonstrate that the lower H_2_ threshold for the growth of *Methanocaldococcus* species at 70–82°C is 17–23 μM (Ver Eecke et al., 2012) and that H_2_-producing hyperthermophilic heterotrophs can support the growth of pure *Methanocaldococcus* strains in the absence of added H_2_ (Bonch-Osmolovskaya and Stetter, 1991; Canganella and Jones, 1994; Muralidharan et al., 1997; Johnson et al., 2006; Ver Eecke et al., 2012). However, there are no reports of H_2_ syntrophy-driven methanogenesis within natural subseafloor microbial communities at thermophilic or hyperthermophilic temperatures. Other factors may also limit the growth of high-temperature methanogens *in situ*, e.g., nitrogen availability (Mehta and Baross, 2006; Ver Eecke et al., 2013), vitamins, or specific trace metal requirements as observed in terrestrial environments (Ünal et al., 2012). In some terrestrial anoxic environments, CH_4_ formation is also inhibited when SO_4_^2-^ concentrations are high (Lovley and Goodwin, 1988). Mesophilic sulfate-reducing bacteria have lower H_2_ half-saturation constants for H_2_ uptake and growth than mesophilic methanogens (Kristjansson et al., 1982; Lovley et al., 1982; Robinson and Tiedje, 1984; Karadagli and Rittmann, 2005). This enables sulfate reducers to inhibit methanogen growth by lowering the partial pressure of H_2_ to concentrations below levels that methanogens can use for growth.

The purpose of this study was to determine, among natural assemblages of thermophilic and hyperthermophilic methanogens, if methanogenesis at hydrothermal vents is limited primarily by the availability of H_2_; if methanogenesis is stimulated by the addition of NH_4_^+^; and if H_2_ syntrophy occurs when natural assemblages of thermophiles and hyperthermophiles are provided only with organic compounds as an energy source. Twenty low-temperature hydrothermal fluids and two nearby background seawater samples were collected from Axial Seamount. Time series samples were collected between and after the April 2011 and April 2015 volcanic eruptions at the site, and sampling included low-temperature vent sites formed by cooling lava flows from the eruptions. These field experiments and subsequent pure culture experiments demonstrate that thermophilic and hyperthermophilic methanogens are generally limited *in situ* by the availability of H_2_, and that H_2_ syntrophy can occur but is more likely at hyperthermophilic growth temperatures.

## Materials and Methods

### Field Sampling

**FIGURE 1:**
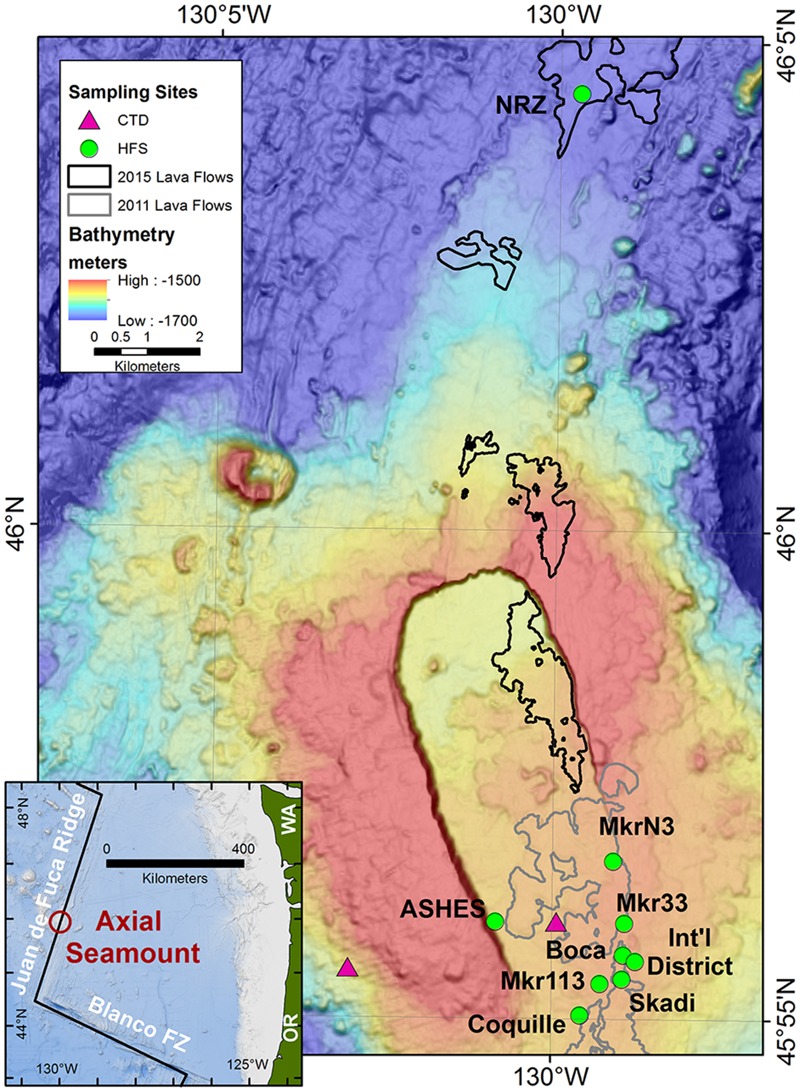
**Map of Axial Seamount and the sample locations.** The hydrothermal sampling sites were along the southeastern rim of the caldera, the western rim of the caldera (ASHES), and 10 km north of the caldera along the North Rift Zone (NRZ). Background seawater was collected 3 km west of the caldera at 1500 m depth and 25 m above the center of the caldera. The outlines of the 2011 and 2015 lava flows are from Caress et al. (2012) and W. Chadwick, personal communication (2016). The inset shows the location of Axial Seamount in the NE Pacific Ocean.

In August 2012, September and October 2013, August 2014, and August 2015, 7–40°C diffuse hydrothermal fluids were collected from 10 vent sites at 1515–1716 m depths from Axial Seamount on the Juan de Fuca Ridge (**Figure 1**). Descriptions of the fluid sample temperatures and the sample sites are provided in Supplementary Table S1. The fluid samples were drawn into 650 ml Tedlar plastic bags with polyethylene valves within rigid housings using the NOAA Hydrothermal Fluid and Particle Sampler (Butterfield et al., 2004). The sampler pumped vent fluid through a titanium nozzle and recorded the temperature of the fluid within the intake nozzle once every second during pumping. Samples were collected using the research submarines *Jason* II and *ROPOS*. Background seawater was collected by shipboard hydrocasts at 1500 m depth directly over the caldera (25 m above the bottom) and 3 km west of the summit with 10 L Niskin bottles (**Figure 1**). The hydrothermal fluid and background seawater samples were divided for cultivation-dependent Most Probable Number (MPN) concentration estimates of thermophiles and hyperthermophiles (100 ml), microcosm incubations (400 ml), and total cell counts (40 ml). All operations at sea occurred on the research vessels *Marcus G. Langseth, Thomas G. Thompson, Falkor*, and *Ronald H. Brown*.

### Microcosm Incubations

For each sample site, 25 ml of hydrothermal fluid or background seawater was added without exposure to air to each of 16 sealed 60 ml serum bottles that had been pre-flushed with either H_2_:CO_2_ (80%:20%) or N_2_:CO_2_ (80%:20%), depending on the headspace composition used for incubation (**Table 1**). The bottles were divided into four sets of four bottles with a pair of bottles from each set incubated at 55°C and 80°C for up to a week or until visibly turbid. Three of the four sets of microcosms (sets A–C) were incubated each of the four study years. Set A was flushed and filled with 200 kPa of H_2_:CO_2_ yielding an estimated aqueous H_2_ concentration of 1.2 mM at their incubation temperatures based on calculations using the geochemical prediction software Geochemist’s Workbench. Sets B and C were flushed and filled with 200 kPa of N_2_:CO_2_, and half of these bottles (set B) were given 1 ml of H_2_:CO_2_ in exchange for 1 ml of N_2_:CO_2_ to produce an estimated aqueous H_2_ concentration of 20 μM at their incubation temperatures. In 2012 and 2013, the remaining four serum bottles (set D) were amended with 4.7 mM NH_4_Cl (2012 only) or 47 μM NH_4_Cl (2013 only) and flushed and filled with 200 kPa of H_2_:CO_2_ to test for growth stimulation by ammonium. The NH_4_Cl concentration was based on that added to our defined methanogen growth medium (see below). In 2014 and 2015, the remaining four serum bottles (set E) were amended with 0.5% (wt vol^-1^) tryptone plus 0.01% (wt vol^-1^) yeast extract and flushed and filled with 200 kPa of N_2_:CO_2_ to test for H_2_ syntrophy. All samples were reduced with 0.025% (wt vol^-1^) each of cysteine-HCl and Na_2_S ⋅9H_2_O. Growth of methanogens was determined by analyzing for CH_4_ in the headspace using gas chromatography once the cells in the bottle had reached stationary growth phase. In 2015, an aliquot of the 80°C and 55°C tryptone/no H_2_ samples (set E) that showed CH_4_ production were filtered onto 0.2-μm pore size nucleopore filters prestained with Irgalan black (Sterlitech, Kent, WA, USA), stained with acridine orange (Francisco et al., 1973), and examined using epifluorescence microscopy. In 2015, the 80°C and 55°C tryptone/no H_2_ samples from the Marker 113 vent site were also separately filtered through Sterivex GP 0.22 μm sterile filter units (Millipore, Billerica, MA, USA) and frozen at -80°C until analyzed. In 2015, 10 ml of hydrothermal fluid was added to sealed Balch tubes without exposure to air, amended separately with 0.1% (wt vol^-1^) sodium formate and 0.5% (wt vol^-1^) sodium acetate, flushed and filled with 200 kPa N_2_:CO_2_, and incubated in duplicate at 80°C and 55°C for up to seven days to determine if these substrates can support methanogenesis at high temperatures.

**Table 1:** Description of microcosms.

Group	Years	Description
Set A (high H_2_)	All	200 kPa H_2_:CO_2_ (80%:20%)
Set B (low H_2_)	All	200 kPa N_2_:CO_2_ (80%:20%), 1 ml of headspace replaced with 1 ml of H_2_:CO_2_
Set C (no H_2_)	All	200 kPa N_2_:CO_2_
Set D (high H_2_ + NH_4_^+^)	2012–2013	200 kPa H_2_:CO_2_ plus 4.7 mM NH_4_Cl (2012) or 47 μM NH_4_Cl (2013)
Set E (no H_2_, tryptone added)	2014–2015	200 kPa N_2_:CO_2_ plus 0.5% tryptone and 0.01% yeast extract

*Each 60 ml serum bottle contained 25 ml of low-temperature diffuse hydrothermal fluid that was incubated in pairs at 55°C and 80°C.*

Total cell counts in the original hydrothermal fluids were done by preserving in duplicate 18 ml of hydrothermal fluid with 1.8 ml of 37% formaldehyde. Samples were stored at 4°C for less than a month prior to counting by epifluorescence microscopy as described above.

### DNA Extraction and 16S rRNA Amplicon Sequencing

In this study and elsewhere (Butterfield et al., 2004; Mehta and Baross, 2006; Ver Eecke et al., 2012, 2013; Fortunato and Huber, 2016), Marker 113 vent showed the highest concentrations of methanogens and methanogenesis at Axial Seamount. Therefore, DNA from each 2015 Marker 113 microcosm that had been amended with tryptone (i.e., set E) and concentrated with a Sterivex filter was extracted and eluted using the MoBio PowerWater DNA extraction kit (MoBio, Carlsbad, CA, USA) as described by the manufacturer to determine which methanogens and other microorganisms were present following the microcosm incubations. The DNA was quantified using a Nanodrop 2000 spectrophotometer (Thermo Scientific, Wilmington, DE, USA) and stored at -20°C. The v4v5 regions of the 16S rRNA gene were amplified separately for bacteria and archaea and prepared for Illumina sequencing from the DNA extractions. Bacterial amplification was carried out as previously described (Huse et al., 2014). The archaeal v4v5 16S rRNA gene was targeted by a combination of five forward primer variants (517F; GCCTAAAGCATCCGTAGC, GCCTAAARCGTYCGTAGC, GTCTAAAGGGTCYGTAGC, GCTTAAAGNGTYCGTAGC, GTCTAAARCGYYCGTAGC) and a single reverse primer (958R; CCGGCGTTGANTCCAATT). Amplification primers were designed based on information from probeBase (Alm et al., 1996; Loy et al., 2003; Huber et al., 2007) and the SILVA database (Ludwig et al., 2004). 16S rRNA amplicon sequencing was performed using an Illumina MiSeq Benchtop sequencer (Illumina, San Diego, CA, USA) at the Marine Biological Laboratory in Woods Hole, MA as described on the Visualization and Analysis of Microbial Population Structures (VAMPSs) website^1^. Paired-end sequences were assessed for quality and merged using the code base previously described (Eren et al., 2013). The sequences were binned into operational taxonomic units (OTUs) using subsampled open reference OTU picking method at 97% sequence identity based on the Greengenes database and taxonomies assigned using the RDP Classifier (Wang et al., 2007) with minimum confidence score 0.8 in QIIME (Caporaso et al., 2010). Sequences are available at the NCBI Sequence Read Archive under accession number SRP071807.

### Media Used

The defined methanogen growth medium for laboratory experimentation and MPN analyses was a modification of DSM 282 medium (Jones et al., 1983; Ver Eecke et al., 2012), which contained (per liter in ddH_2_O): 0.14 g of K_2_HPO_4_, 0.14 g of CaCl_2_ ⋅7H_2_O, 0.25 g of NH_4_Cl, 3.4 g of MgSO_4_ ⋅7H_2_O, 5.1 g of MgCl_2_ ⋅6H_2_O, 0.34 g of KCl, 0.05 mg of NiCl_2_ ⋅6H_2_O, 0.05 mg of Na_2_SeO_3_ ⋅5H_2_O, 30 g of NaCl, 1 g of NaHCO_3_, 1 g of NaS_2_O_3_, 0.24 g of Na_2_MoO_4_ ⋅2H_2_O, 10 ml of Wolfe’s minerals, 10 ml of Wolfe’s vitamins, and 0.25 mg of resazurin. For the 2012 MPNs, 0.24 g of Na_2_MoO_4_ ⋅2H_2_O was also added to suppress sulfate reduction but was omitted in subsequent years. The medium was pH balanced to 6.0, reduced with 0.025% each of cysteine-HCl and Na_2_S ⋅9H_2_O, and pressurized with 200 kPa of H_2_:CO_2_ headspace. The autotrophic sulfur-reducer medium was the same as the methanogen medium except that 10 g l^-1^ of elemental sulfur were added and the medium was reduced with 3.2 mM dithiothreitol (DTT). The heterotroph medium for MPN estimates was based on the Adams medium (Adams et al., 2001) and contained 0.5% tryptone plus 0.01% yeast extract. It was pH balanced at 6.8, reduced with 0.025% each of cysteine-HCl and Na_2_S ⋅9H_2_O, and pressurized with 100 kPa of N_2_:CO_2_ headspace. The heterotroph-methanogen co-culture medium was the modified DSM 282 medium with 0.1 ml of 10 mM Na_2_WO_4_ ⋅2H_2_O, 1 ml of 0.2% (NH_4_)_2_Fe(SO_4_)_2_-(NH_4_)_2_Ni(SO_4_)_2_, and 0.5% tryptone plus 0.01% yeast extract added with 200 kPa of N_2_:CO_2_ headspace. The medium was pH balanced to 6.8.

### Most Probable Number (MPN) Cell Estimates

Three-tube MPN analyses were used by adding 3.3, 0.33, and 0.03 ml of the hydrothermal fluid samples in triplicate to the methanogen, autotrophic sulfur reducer, and heterotroph media as previously described (Ver Eecke et al., 2009). After inoculation, the tubes were incubated at 80°C and 55°C for up to 7 days. Growth in the tubes was confirmed using phase-contrast light microscopy. Growth of methanogens and H_2_-producing heterotrophs was verified by analyzing all of the tubes for CH_4_ and H_2_, respectively, in the headspace using gas chromatography. Total and H_2_-producing heterotroph cell concentration estimates were scored and reported separately based on tubes that had cells versus those with H_2_. In order to estimate the concentration of non-methanogenic autotrophs in the autotrophic sulfur medium, the estimated number of methanogens in the autotrophic sulfur medium MPN tubes was subtracted from the estimated concentration of total cells.

### Pure and Co-culture Growth Conditions

*Methanocaldococcus bathoardescens* JH146 (DSM 27223; Ver Eecke et al., 2013; Stewart et al., 2015), *Methanothermococcus* sp. strain BW11 (DSM 100453; Stewart et al., 2016), and *Thermo-coccus paralvinellae* ES1 (DSM 27261; Pledger and Baross, 1989; Hensley et al., 2014, 2016) were used for pure and co-culture experiments from our hyperthermophile culture collection. *Methanocaldococcus jannaschii* JAL-1 (DSM 2661; Jones et al., 1983) and *Methanothermococcus thermolithotrophicum* (DSM 2095; Huber et al., 1982) were purchased from the Deutsche Sammlung von Mikrooganismen und Zellkulturen GmbH (DSMZ, Braunschweig, Germany).

*Methanocaldococcus jannaschii* and *M. bathoardescens* were grown at 80°C and *M. thermolithotrophicum* and *Methano-thermococcus* sp. strain BW11 were grown at 55°C in 25 ml of modified DSM 282 methanogen medium in 60 ml serum bottles with 200 kPa of H_2_:CO_2_ for up to 5 days to compare their maximum CH_4_ production amounts with those of the field microcosms. *M. jannaschii* and *M. thermolithotrophicum* were grown at 82°C and 65°C, respectively, in the methanogen medium described above minus cysteine and all other sources of nitrogen with varying concentrations of NH_4_Cl to determine the effect of nitrogen availability. *M. jannaschii* and *M. bathoardescens* were also grown at 82°C in Balch tubes in modified DSM 282 medium without added vitamins following five transfers on vitamin-free medium to determine the effect of vitamins on their growth.

For each growth kinetic experiment, 18 Balch tubes containing growth medium were inoculated simultaneously with a logarithmic growth phase culture that had been transferred three times on that medium and incubated in a forced-air incubator. Three tubes were permanently removed from the incubator at various time points. The cell concentration in each tube was determined using a Petroff–Hausser counting chamber and phase contrast light microscopy. The growth rate (μ) of the culture was determined by fitting an exponential curve to the growth data. The total amount of CH_4_ in each tube that had been cooled to room temperature was determined by measuring the volume of gas in each tube and the amount of CH_4_ in 100 μl of headspace using gas chromatography. The CH_4_ production yield (Y_p/x_) was determined from the slope of the amount of CH_4_ per tube plotted against the total number of cells per tube. The rate of CH_4_ production per cell is calculated from Y_p/x_ × μ/0.693 as previously described (Ver Eecke et al., 2013). The 95% confidence intervals for growth and CH4 production rates were calculated as previously described (Zar, 1996).

*Thermococcus paralvinellae* was grown separately on the co-culture base medium described above with either 0.5% tryptone plus 0.01% yeast extract; 0.5% maltose plus 0.01% yeast extract; or 0.5% each of tryptone and maltose plus 0.01% yeast extract media, each with 200 kPa of N_2_:CO_2_ headspace, at 82°C and 60°C to determine how temperature affects its rate of H_2_ production on various substrates. The rate of H_2_ production was measured as described above for the rate of CH_4_ production by the methanogens. For the co-culture experiments, *T. paralvinellae* was grown alone at 82°C and 60°C, in co-culture with *M. bathoardescens* at 82°C, and in co-culture with *Methanothermococcus* sp. strain BW11 at 60°C in 160 ml serum bottles containing 50 ml of modified DSM 282 medium supplemented with 0.5% each of maltose and tryptone plus 0.01% yeast extract with 200 kPa of N_2_:CO_2_ headspace. The heterotrophs and methanogens were combined during inoculation in a 10:1 cell ratio. The co-culture was established immediately and did not require prior co-culture transfers. At various time points during growth, the amount of H_2_ and CH_4_ was measured from triplicate incubation bottles using gas chromatography.

## Results

### MPN Cell Estimates in Hydrothermal Fluids

In 2012, the concentrations of all thermophiles and hyperthermophiles in all samples were very low compared to the concentrations in subsequent years (**Table 2** and Supplementary Table S2). In 2013, methanogens that grew at 80°C were detected in low-temperature hydrothermal fluids at Marker 113, Marker 33, ASHES, Boca, and Skadi. They were not detected in vent fluids from Coquille, Marker N3 or the International District (**Table 2** and Supplementary Table S2). Methanogens that grew at 55°C were found at lower concentrations at Marker 113, Marker 33, ASHES, Boca, Skadi, Marker N3, and Coquille, but were not detected at the International District (**Table 2** and Supplementary Table S2). Heterotrophs that grew at 80°C and 55°C were present in relatively high concentrations at each vent site in 2013 (**Table 2** and Supplementary Table S2). The concentrations of heterotrophs that produced H_2_ were lower at 330–7,200 cells L^-1^ at 80°C, and only Marker 113 and Boca showed any H_2_ producing heterotrophs at 55°C, which were at low concentrations (120–270 cells L^-1^). The heterotrophs that grew at 80°C were all coccoids, while those that grew at 55°C were predominantly rods. Non-methanogenic autotrophs that grew at 80°C and 55°C were also present at most of the vent sites in 2013 (**Table 2** and Supplementary Table S2).

**Table 2:** Most-probable number (MPN, L^-1^) estimates of heterotrophs, H_2_-producing heterotrophs, methanogens, and non-methanogenic hydrogenotrophs that grow at 55°C and 80°C.

	80°C				55°C			
	2012	2013	2014	2015	2012	2013	2014	2015
**Marker 113**								
Heterotrophs	2,790	>33,000	>33,000	1,140	ND*^a^*	>33,000	>33,000	1,140
H_2_-prod. heterotrophs	220	330	33,000	ND	ND	120	2,250	276
Methanogens	120	1,050	6,300	1,140	ND	330	13,800	33,000
Other autotrophs	102	5,220	ND	ND	270	5,610	1,350	ND
Initial total cells (×10^8^, L^-1^)*^b^*	3.4	5.4	8.5	15.0				
**Marker 33**								
Heterotrophs	–	>33,000	>33,000	33,000	–	>33,000	>33,000	1,290
H_2_-prod. heterotrophs	–	7,200	4,500	ND	–	ND	840	108
Methanogens	–	13,800	13,800	1,290	–	330	2,790	ND
Other autotrophs	–	ND	ND	33,000	–	32,310	4,224	13,800
Initial total cells (×10^8^, L^-1^)	–	2.8	9.6	8.1				
**Anemone (ASHES)**								
Heterotrophs	7,200	13,800	>33,000	>33,000	690	7,200	>33,000	13,800
H_2_-prod. heterotrophs	2,790	1,290	1,290	120	ND	ND	13,800	450
Methanogens	ND	276	1,290	1,290	ND	690	120	450
Other autotrophs	276	1,170	2,340	13,680	270	2,670	4,500	4,380
Initial total cells (×10^8^, L^-1^)	0.8	4.1	9.4	10.0				

*^a^ND, not detected. *^b^*Total cell concentration is for the hydrothermal fluid sample prior to incubation.*

The Marker 113, Marker 33, and ASHES vent sites were selected for time series measurements in 2014 and 2015 (**Table 2**). During those years, methanogens that grew at 80°C were found at each site. From 2012 to 2015, methanogens that grew at 55°C increased in abundance from not detectable to 33,000 cells L^-1^ at Marker 113, were not detectable at Marker 33 in 2015, and were consistently present at relatively low concentrations at ASHES. Heterotrophs that grew at 80°C and 55°C were often present in high concentrations, but in 2015 decreased significantly in concentration at 80°C at Marker 113 and at 55°C at Marker 113 and Marker 33. The concentrations of H_2_-producing heterotrophs that grew at 80°C was relatively high at all three vents in 2014 but decreased significantly in 2015. Similarly, H_2_-producing heterotrophs that grew at 55°C were higher in concentration at the three vents in 2014 than in 2015. As in 2013, the heterotrophs that grew at 80°C were all coccoids, while those that grew at 55°C were predominantly rods. From 2013 to 2015, non-methanogenic autotrophs decreased in concentration at 80°C and 55°C at Marker 113 until they were no longer detectable, increased in concentration at 80°C at Marker 33, and remained relatively constant at ASHES. At the North Rift Zone (NRZ) eruption site in 2015, there were 2,790 methanogens L^-1^ that grew at 80°C and 33,000 methanogens L^-1^ that grew at 55°C (Supplementary Table S2). No non-methanogenic autotrophs grew at either 80°C or 55°C from NRZ fluids. No methanogens, heterotrophs or non-methanogenic autotrophs grew at either 80°C or 55°C from background seawater collected at 1,500 m depth 3 km away from the seamount summit or 25 m above the summit caldera, with the exception of 90 heterotrophs L^-1^ that grew at 55°C from over the caldera (Supplementary Table S2).

### Growth in Microcosms on H_2_, CO_2_, and NH_4_^+^

**FIGURE 2:**
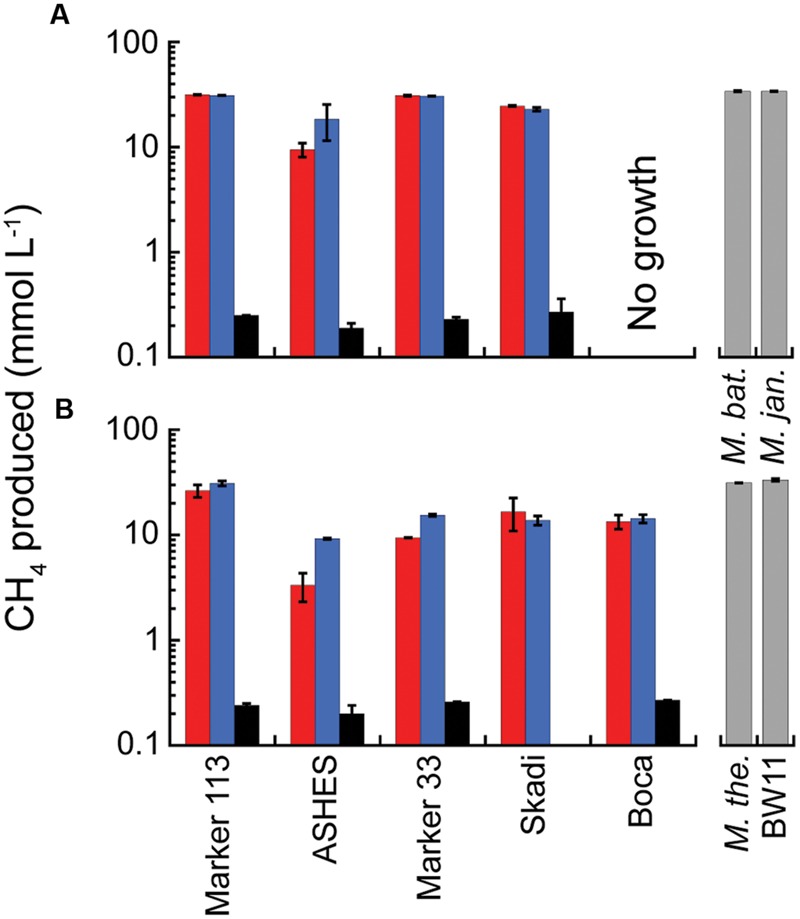
**Average total CH_4_ production in 2013 microcosms.** The microcosms were incubated at 80°C **(A)** and 55°C **(B)** and amended with 200 kPa of H_2_:CO_2_ (red); 200 kPa of H_2_:CO_2_ plus 47 μM NH_4_Cl (blue); and 2 kPa of H_2_ and 198 kPa of N_2_:CO_2_ (black). The gray columns show the total CH_4_ production for the four pure cultures in modified DSM 282 medium for comparison. The sample bars represent the range of the duplicate incubations.

In 2012, consistent with the MPN concentration estimates, no CH_4_ was detected in any of the microcosms at either 80° or 55°C, except for one high H_2_ microcosm and one low H_2_ microcosm incubated at 80°C from Marker 113. In 2013, CH_4_ production occurred in microcosms amended with H_2_, CO_2_ and NH_4_Cl at 80°C in hydrothermal fluids from Marker 113, ASHES, Marker 33, and Skadi with up to 31.6 mmol CH_4_ produced L^-1^ of vent fluid (**Figure 2A**). Methanogenesis also occurred in microcosms at 55°C in fluids from the same sites plus Boca vent with up to 31.0 mmol CH_4_ produced L^-1^ (**Figure 2B**). The amount of CH_4_ produced when only 1 ml of H_2_:CO_2_ (20 μM H_2_) was added to each bottle was 1–3% the amount of CH_4_ produced when 200 kPa of H_2_:CO_2_ were added (**Figure 2**). The amount of CH_4_ produced when the microcosms were amended with 47 μM NH_4_Cl in addition to 200 kPa of H_2_:CO_2_ was generally the same as the amount of CH_4_ produced when only 200 kPa of H_2_:CO_2_ were added, with the exceptions of the microcosms from ASHES at both incubation temperatures and from Marker 33 incubated at 55°C (**Figure 2**). Consistent with the MPNs, there was no methanogenesis at 80°C and 55°C in hydrothermal fluids from Marker N3 and the International District, nor in either background seawater sample. There was no CH_4_ in any 80°C or 55°C microcosms amended only with N_2_:CO_2_. For comparison, the total amounts of CH_4_ produced by *M. bathoardescens* and *M. jannaschii* grown to stationary growth phase at 82°C in modified DSM 282 methanogen medium were the same as the 80°C microcosms (**Figure 2A**). Similarly, the total amounts of CH_4_ produced by *M. thermolithotrophicum* and *Methanothermococcus* sp. strain BW11 at 55°C were the same as the 55°C microcosms (**Figure 2B**).

**FIGURE 3:**
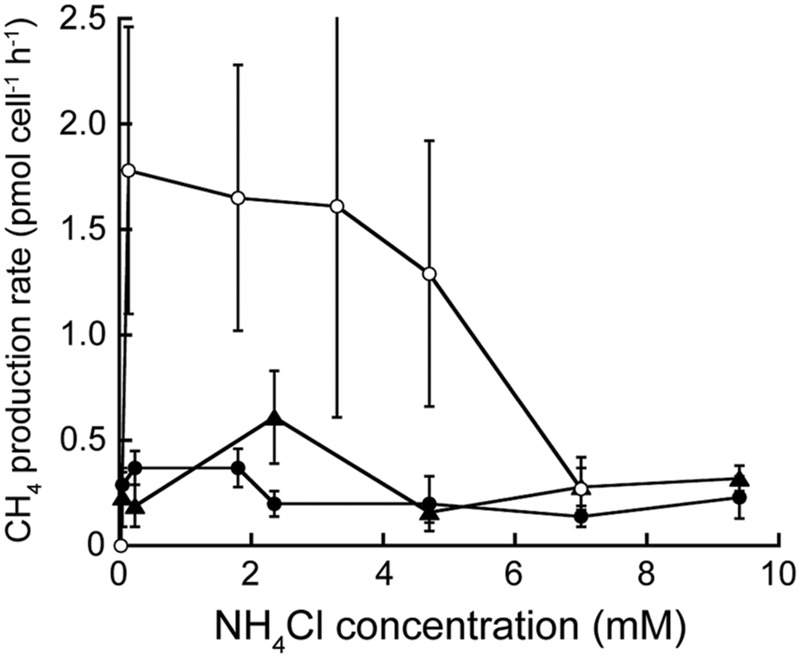
**Cell-specific rate of CH_4_ production at varying NH_4_Cl concentrations.** *Methanocaldococcus jannaschii* (●) and *M. bathoardescens* (○) were grown at 82°C, and *M. thermolithotrophicum* (▲) was grown at 65°C. The data for *M. bathoardescens* are from Ver Eecke et al. (2013) and are provided for comparison. The error bars represent the 95% confidence intervals.

*Methanothermococcus jannaschii* grown at 82°C and *M. thermolithotrophum* grown at 65°C at varying NH_4_Cl concentrations in otherwise nitrogen-free medium did not show any change in cell specific CH_4_ production rate in medium with 47 μM to 9.4 mM NH_4_Cl added (**Figure 3** and Supplementary Table S3). Furthermore, the growth rates of *M. jannaschii* and *M. bathoardescens* grown at 82°C without vitamins were 1.19 h^-1^ ± 0.32 h^-1^ (±95% confidence interval) and 2.74 h^-1^ ± 1.01 h^-1^, respectively, and were not significantly different than the rates for each organism with added vitamins.

### H_2_ Syntrophy in Microcosms

**FIGURE 4:**
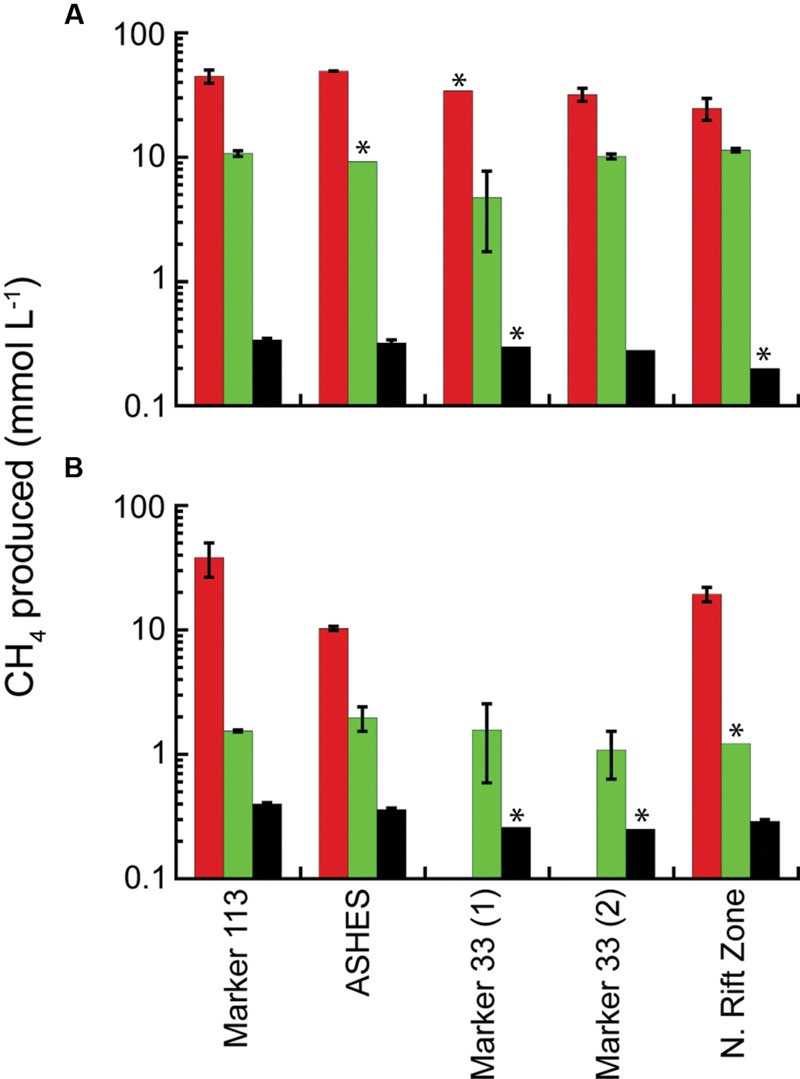
**Average total CH_4_ production in 2015 microcosms.** The microcosms were incubated at 80°C **(A)** and 55°C **(B)** and amended with 200 kPa of H_2_:CO_2_ (red); 200 kPa of N_2_:CO_2_, 0.5% tryptone and 0.01% yeast extract (green); and 2 kPa of H_2_ and 198 kPa of N_2_:CO_2_ (black). The sample bars represent the range of the duplicate incubations. The asterisks show where there was growth in only one microcosm bottle.

In 2014, methanogenesis occurred in microcosms amended with 200 kPa of H_2_:CO_2_ or separately with tryptone plus N_2_:CO_2_ at 80°C and 55°C in hydrothermal fluids from Marker 113, ASHES, and Marker 33 with up to 9.5 mmol CH_4_ produced L^-1^ (Supplementary Figure S1). The amount of CH_4_ produced was lower and less consistent than observed in 2013 and 2015. In contrast, in 2015 methanogenesis occurred at 80°C in hydrothermal fluids from Marker 113, ASHES, Marker 33, and the NRZ eruption site with up to 49.3 mmol CH_4_ produced L^-1^ (**Figure 4A**). Methanogenesis occurred at 55°C in hydrothermal fluids from Marker 113, ASHES, and the NRZ with up to 38.2 mmol CH_4_ produced L^-1^ (**Figure 4B**). Similar to MPN observations, there was no methanogenesis at 55°C in two separate sets of microcosms containing fluid from Marker 33 that were amended only with 200 kPa of H_2_:CO_2_ (**Figure 4B**). As seen in 2013, the amount of CH_4_ produced in 2015 when only 1 ml of H_2_:CO_2_ was added to each bottle was 1–3% the amount of CH_4_ produced when 200 kPa of H_2_:CO_2_ were added (**Figure 4**). The amount of CH_4_ produced when microcosms were amended with tryptone plus N_2_:CO_2_ was 4.7–11.4 mmol L^-1^ at 80°C and was less (1.1–2.0 mmol L^-1^) at 55°C (**Figure 4**). Microscopic observations of the 2015 tryptone plus N_2_:CO_2_ microcosms following incubation show that the 80°C microcosms contain almost all coccoid-shaped cells (Supplementary Figure S2A) while the 55°C microcosms contain mostly rod-shaped cells with some coccoids (Supplementary Figure S2B). There was no CH_4_ in any 80°C or 55°C microcosms amended only with N_2_:CO_2_ or in 80°C and 55°C microcosms amended with tryptone plus N_2_:CO_2_ containing background seawater collected 25 m above the caldera. There also was no CH_4_ in any 80°C and 55°C microcosms amended with either acetate or formate with 200 kPa of N_2_:CO_2_ in the headspace.

**FIGURE 5:**
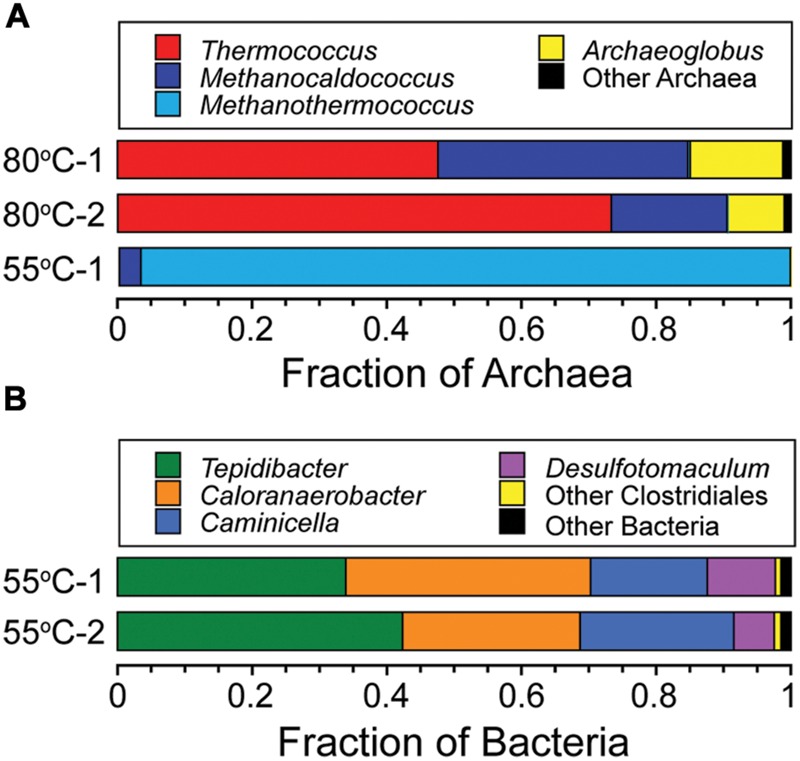
**Phylogenetic diversity of Archaea and Bacteria in the 80°C and 55°C microcosms.** Taxonomic breakdown and relative abundance at the genus level for archaeal **(A)** and bacterial **(B)** 97% 16S rRNA gene OTUs from microcosms following incubation at 80°C and 55°C using diffuse hydrothermal fluids collected from the Marker 113 vent site.

Phylogenetic analysis showed that DNA from microcosms incubated at 80°C only amplified with archaeal primers. Microcosms incubated at 55°C amplified with bacterial primers but only one of the replicates amplified with archaeal primers. Sequencing depths ranged from 78,143 to 163,507 sequences, with a mean of 114,736 reads per sample. Rarefraction analysis showed that sequencing efforts were sufficient to represent the diversity of the samples examined. Archaeal sequence reads were binned into 161 OTUs based on 97% sequence identity after singletons were removed. Archaeal sequences in the 80°C microcosms belonged to genera *Thermococcus* (46–73% of sequences), *Methanocaldococcus* (17–37% of sequences), and *Archaeoglobus* (9–14% of sequences) with some sequences that belong to *Methanothermococcus, Palaeococcus*, and *Nitrosopomilus* (**Figure 5A**). Archaeal sequences observed in 55°C microcosm were dominated by the genera *Methanothermococcus* (96% of sequences) and *Methanocaldococcus* (3% of sequences) (**Figure 5A**). Bacterial sequence reads were binned into 188 OTUs based on 97% sequence identity after singletons were removed. The sequences were dominated by the genera *Tepidibacter* (34–42% of sequences), *Caloranaerobacter* (26–36% of sequences), *Caminicella* (17–23% of sequences), and *Desulfotomaculum* (6–10% of sequences) (**Figure 5B**), which all belong to the order *Clostridiales* (98% of sequences in both replicates).

**FIGURE 6:**
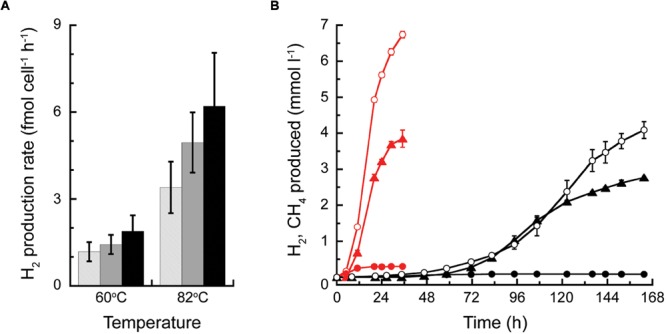
**H_2_ production by *Thermococcus paralvinellae* and CH_4_ production by *M. bathoardescens* and *Methanothermococcus* sp. BW11 grown alone and in co-culture. (A)** Cell-specific H_2_ production rate of *T. paralvinellae* at 82°C and 60°C when grown on 0.5% maltose and 0.01% yeast extract (light gray), 0.5% tryptone and 0.01% yeast extract (gray), and 0.05% each of maltose and tryptone and 0.01% yeast extract (black). **(B)** H_2_ (circles) and CH_4_ (triangles) produced when *T. paralvinellae* was grown alone (open circles) and in co-culture (filled symbols) with *M. bathoardescens* at 82°C (red) and with *Methanothermococcus* sp. BW11 at 60°C (black).

The cell specific rates of H_2_ production by *T. paralvinellae* grown on maltose, tryptone, and combination of maltose and tryptone were not significantly different from each other at either temperature examined (**Figure 6A**). The rates decreased from 3.4 to 6.2 fmol H_2_ cell^-1^ h^-1^ at 82°C to 1.2–1.9 fmol H_2_ cell^-1^ h^-1^ at 60°C. *T. paralvinellae* grown alone at 82°C and 60°C produced up to 6.7 mmol H_2_ L^-1^ at specific production rates of 0.22 h^-1^ and 0.04 h^-1^, respectively (**Figure 6B**). When grown in co-culture with *M. bathoardescens* at 82°C and with *Methanothermococcus* strain BW11 at 60°C, the amount of H_2_ produced remained below 0.3 mmol L^-1^ and 0.1 mmol L^-1^, respectively. The amount of CH_4_ produced in co-culture at 82°C and 60°C was 3.9 mmol L^-1^ and 2.8 mmol L^-1^ and the specific rates of CH_4_ production were 0.16 h^-1^ and 0.06 h^-1^, respectively (**Figure 6B**).

## Discussion

This study demonstrated that naturally occurring methanogens at Axial Seamount are primarily limited by H_2_ availability and not by the availability of other compounds such as nitrogen sources, trace metals, or vitamins. It also showed that methane production can occur among natural assemblages of methanogens by H_2_ syntrophy but appears to be more prevalent at hyperthermophilic temperatures rather than thermophilic temperatures due to the growth of competitors at the lower temperatures.

The eruption cycle at Axial Seamount provided the opportunity to examine the impact of eruption events on methanogenic and heterotrophic microbial communities in hydrothermal fluids there. Previous work at Axial Seamount after eruptive events showed that microbial diversity appears to increase in the year following the eruption, with some vents, including snowblowers, quickly dying out, while other pre-existing vents continue to vent post-eruption (Huber et al., 2002, 2003, 2006a; Ver Eecke et al., 2012; Meyer et al., 2013). In 2012, 16 months after the 2011 eruption, the concentrations of thermophiles, hyperthermophiles and total cells at Marker 113, ASHES, and Boca were lower than at any other time during the sampling time series suggesting there is a period of microbial quiescence between eruptions that is widespread throughout the caldera. In 2014, as the magma chamber inflated leading up to the 2015 eruption (Kelley et al., 2015), the concentrations of cultivable hyperthermophilic and thermophilic methanogens and heterotrophs reached their highest points at Marker 113 and Marker 33 on the eastern flank of the caldera, close to the eruptive fissures and the underlying magma chamber, but remained relatively constant at ASHES on the western flank of the caldera. Methanogen and heterotroph concentrations decreased at Marker 113 and Marker 33 in 2015 4 months after the eruption. This suggests that the eastern flank of Axial Seamount may be heading toward another period of microbial quiescence within the anoxic subseafloor. The exception was at Marker 113 where methanogens that grew at 55°C increased in concentration through 2015 while other autotrophs that grew at 55°C decreased in concentration until they were undetectable. In contrast, CH_4_ production in microcosms was lower in 2014 than in 2013 and 2015 suggesting that the elevated concentrations of cultivable methanogens may have partially depleted some growth factor in the system other than H_2_ that year which led to poorer growth in the microcosms. As previously observed at Boca following the 2011 eruption (Meyer et al., 2013), methanogens were relatively abundant in hydrothermal fluids emanating from new basalt flows on the North Rift Zone (NRZ) caused by the 2015 eruption. No other thermophilic or hyperthermophilic hydrogenotrophs were found at the site.

Low-temperature hydrothermal fluids at Axial Seamount (e.g., <40°C) are typically depleted or nearly depleted in H_2_ (e.g., <3 μmol kg^-1^) due to microbial H_2_ consumption or low-H_2_ source fluids (Ver Eecke et al., 2012). In these environments, H_2_ syntrophy may serve as an alternative source of H_2_ to help sustain methanogens and other hydrogenotrophs. Mesophilic sulfide-oxidizing bacteria, abundant macrofauna, and seawater ingress into hotter hydrothermal environments may provide the labile organic compounds necessary to support H_2_-producing heterotrophs. The predominant heterotrophs at hyperthermophilic temperatures in low-temperature fluids at Axial Seamount are *Thermococcus* species (Huber et al., 2002, 2006a). All *Thermococcus* species possess at least one hydrogenase (Schut et al., 2012), and some have as many as seven hydrogenases (Lee et al., 2008; Jung et al., 2014). In this study, *T. paralvinellae*, which possesses the genes for seven hydrogenases (Jung et al., 2014), produced H_2_ from protein and carbohydrate substrates at equal rates that both increased with increasing temperature. Microcosm incubations at both 80°C and 55°C demonstrated that it is H_2_ and not formate or acetate that is used by the methanogens at high temperatures. This is likely due to the lower energy yield for methanogenesis using formate and acetate as carbon and energy sources (Deppenmeier, 2002). *Thermococcus* is widely representative of H_2_ producers in many diverse subseafloor ecosystems. They were the only archaeal 16S rRNA gene sequences found 99 and 194 meters below the seafloor (mbsf) in Nankai Trough sediments (Kormas et al., 2003). They dominated the archaeal 16S rRNA gene diversity of a sediment horizon collected 634 mbsf in the Canterbury Basin (Ciobanu et al., 2014) and in 80–90°C water-flooded oil reservoirs in the Sinopec Shengli oil field (Junzhang et al., 2014). They are commonly found in ridge flanks basement outcrops (Huber et al., 2006b; Ehrhardt et al., 2007) and petroleum reservoirs (Stetter et al., 1993; L’Haridon et al., 1995; Miroshnichenko et al., 2001; Dahle et al., 2008). Therefore, *Thermococcus* may have the potential to degrade local organic compounds and provide H_2_ to collocated hydrogenotrophic microbes in non-hydrothermal vent subseafloor anoxic environments as well.

The taxonomic analysis of microcosm incubations demonstrates a transition from hyperthermophilic archaeal H_2_ syntrophy to thermophilic bacterial H_2_ syntrophy with decreasing incubation temperature. In 55°C microcosms, the amount of CH_4_ produced through syntrophy decreased relative to the 80°C microcosms, suggesting that H_2_ syntrophy-driven methanogenesis may be more pronounced at hyperthermophilic temperatures. The 80°C microcosms produced the most CH_4_ and only archaeal DNA was amplified from these incubations, including the H_2_-producing heterotroph *Thermococcus* and the H_2_-consuming methanogens *Methanocaldococcus* and *Methanothermococcus* species. These were the predominant organisms found in previous Marker 113 fluids incubated at 80°C with H_2_ and bicarbonate for stable-isotope probing analysis (Fortunato and Huber, 2016) and other hyperthermophile culture- and molecular-based analyses (Ver Eecke et al., 2012). In contrast, the 55°C microcosms produced significantly less CH_4_, bacterial DNA was amplified in both samples, and archaeal DNA was amplified in only one sample. The predominant bacteria found in these samples were most closely related to the genera *Tepidibacter, Caloranaerobacter*, and *Caminicella*. Each of these genera is a thermophilic member of the *Clostridia* and has representatives that were isolated from hydrothermal vents that ferment peptides and carbohydrates and produce H_2_, CO_2_, carboxylic acids, alcohols, and alanine (Wery et al., 2001; Alain et al., 2002; Slobodkin et al., 2003; Jiang et al., 2014). Among hydrogenotrophs, *Methanothermococcus* was the predominant archaeon sequence found in one 55°C microcosm, and sequences most closely related to *Desulfotomaculum* were also found among the bacteria. *Desulfotomaculum thermosubterraneum* is a thermophilic sulfur reducer that can consume H_2_, CO_2_, carboxylic acids, alcohols, and alanine (Kaksonen et al., 2006). These results indicate the capacity of hydrothermal vent microbial communities to perform various forms of H_2_ syntrophy depending on growth temperature.

In order to quantify how H_2_ limitation and syntrophy impact CH_4_ production, primary production, and total biomass within subseafloor environments at hydrothermal vents and elsewhere, it will be necessary to develop models of growth and cell-cell interactions that can be applied to these environments. All models make certain *a priori* assumptions, and this study demonstrated that in most circumstances, the growth of thermophilic and hyperthermophilic methanogens *in situ* is primarily limited by the availability of H_2_ and heat. The ability of some *Methanocaldococcus* and *Methanothermococcus* species to fix N_2_ suggests that they are adapted to low-nitrogen environments (Belay et al., 1984; Mehta and Baross, 2006; Nishizawa et al., 2014). Depleted NO_3_^-^ concentrations in diffuse vent fluids at Axial (Butterfield et al., 2004; Bourbonnais et al., 2012) suggest that NO_3_^-^ may be limited in the subseafloor, although metatranscriptomic analysis of Marker 113 hydrothermal fluids shows that the genes for denitrification are expressed (Fortunato and Huber, 2016). However, ΣNH_3_ in high-temperature source fluids at Axial is variable and reaches 16 μmol kg^-1^ (approximately 1/3 of the ambient deep seawater NO_3_^-^), providing a modest nitrogen source for primary producers. In most of the microcosm incubations in this study, the addition of 47 μM NH_4_Cl did not enhance the production of CH_4_, the amount of CH_4_ produced by natural methanogen assemblages in hydrothermal fluid was the same as those by pure cultures in nutrient-replete medium, and the omission of vitamins to pure cultures had no effect on their growth. Therefore, natural assemblages of thermophilic methanogens do not appear to be limited by nitrogen or trace nutrient requirements in most cases. Methane production was low in the 2014 microcosms relative to 2013 and 2015, despite the fact that the number of cultivable methanogens was relatively high, suggesting that there are periods where methanogens might be at least partially limited in their growth by factors other than H_2_ and heat.

## Conclusion

The microcosm results validate the modeling assumption made in the lab that the artificial conditions generated are generally representative of the growth of natural assemblages of methanogens in a mixture of hydrothermal fluid and seawater. They also define the constraints on methanogenesis at hydrothermal vents and tie together metagenomics and metatranscriptomic data with ecosystem functioning. These will help reveal the physiological state of methanogens *in situ* and assist in the effort to model the rates of methane formation in hydrothermal systems on varying substrates.

## Author Contributions

BT, LS, and JFH designed and performed research, analyzed data, and wrote the paper; DB provided the Hydrothermal Fluid and Particle Sampler for fluid sample collection at the vents; HM designed the archaeal 16S rRNA gene primers; and JAH provided the nucleotide sequence data and served as the Program Leader.

## Conflict of Interest Statement

The authors declare that the research was conducted in the absence of any commercial or financial relationships that could be construed as a potential conflict of interest.

## Footnotes

1 https://vamps.mbl.edu/resources/primers.php
