# Supplementary material for: Hydrogen Limitation and Syntrophic Growth among Natural Assemblages of Thermophilic Methanogens at Deep-sea Hydrothermal Vents
Source: Front Microbiol. 2016 Aug 5;7:1240. doi: 10.3389/fmicb.2016.01240 (PMC4974244; doi:10.3389/fmicb.2016.01240)
Supplement: Supplementary file 1 [file Data_Sheet_1.PDF]

frontiers

Supplementary Material

Hydrogen limitation and syntrophic growth among natural assemblages of thermophilic methanogens at a deep-sea hydrothermal vent

Begüm D. Topçuoğlu, Lucy C. Stewart, Hilary G. Morrison, David A. Butterfield, Julie A. Huber, James F. Holden*

*** Correspondence:** Dr. James F. Holden: jholden@microbio.umass.edu.

Figures S1-S2

Tables S1-S3

1 Supplementary Figures and Tables

1.1 Supplementary Figures

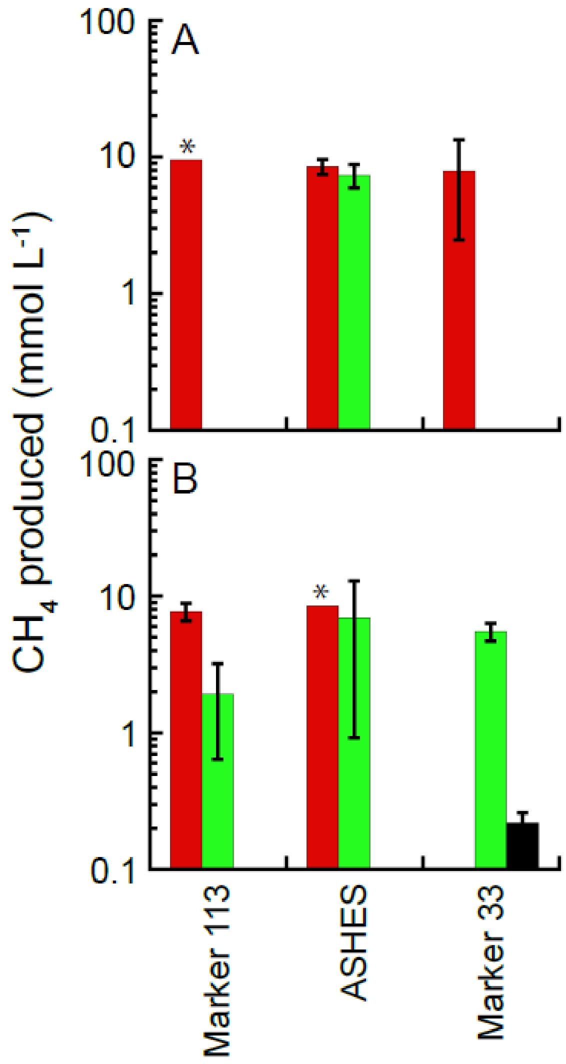

Supplementary Figure 1. Average total CH_4 production in 2014 microcosms. The microcosms were incubated at 80°C (A) and 55°C (B) and amended with 1.6 atm of H_2 and 0.4 atm of CO_2 (red); 1.6 atm of N_2, 0.4 atm of CO_2, 0.5% tryptone and 0.01% yeast extract (green); and 0.02 atm H_2, 1.58 atm of N_2, and 0.4 atm of CO_2 (black). The sample bars represent the range of the duplicate incubations. The asterisks show where there was growth in only one microcosm bottle.

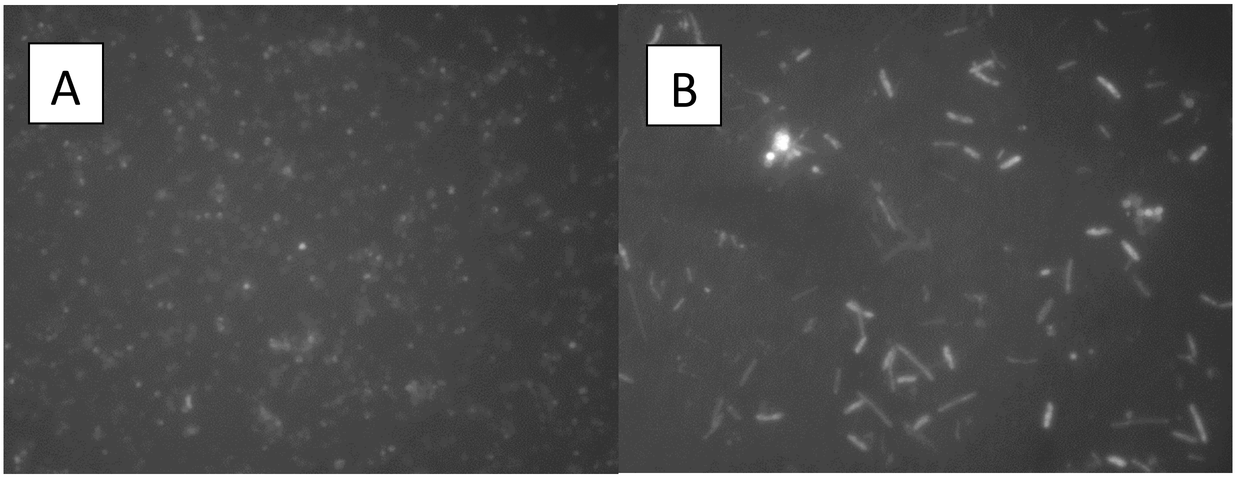

Supplementary Figure 2. Epifluorescence micrographs of Marker 113 cells grown in the microcosms on 0.5% tryptone and 0.01% yeast extract at 55°C (A) and 80°C (B). All of the 55°C microcosm incubations containing organic supplements contained mostly rods, while all of the 80°C microcosm incubations containing organic supplements contained nearly exclusively coccoids. The scale bars are 10 µm.

1.2 Supplemental Tables

Supplemental Table 1. Description of Axial Seamount hydrothermal sampling sites.

Site	Description
Marker 113	Basalt-hosted diffuse hydrothermal vent field on the edge of a lava flow collapse zone (1521 m depth). The temperatures of the diffuse fluids were 28°C in 2012, 24°C in 2013, 25°C in 2014, and 25°C in 2015.
Marker 33	Basalt-hosted diffuse hydrothermal vent field flowing from the cooled 2011 eruptive lava flow (1516 m depth). The temperatures of the diffuse fluids were 27°C in 2013, 20°C in 2014, and 34°C and 40°C in 2015.
Anemone (ASHES)	Diffuse hydrothermal venting emanating from the top of a 0.5 m tall sulfide mound (1543 m depth) within 10 m of the Hell metal sulfide hydrothermal edifice emitting 286-297°C fluid. The temperatures of the diffuse fluids were 29°C in 2012 and 2013, 35°C in 2014, and 20°C in 2015.
Boca	Basalt-hosted diffuse hydrothermal vent field flowing from the cooled 2011 eruptive lava flow (1517 m depth). The temperatures of the diffuse fluids were 10°C in 2012 and 7°C in 2013.
Fuzzy Tubeworm Bush (ASHES)	Basalt-hosted diffuse hydrothermal venting (1544 m depth) within 10 m of the Inferno metal sulfide edifice emitting 319°C fluid. The temperature of the diffuse fluid was 28°C in 2012.
Skadi	Basalt-hosted diffuse hydrothermal vent field on the edge of a lava flow collapse zone within 10 m of a metal sulfide hydrothermal edifice emitting 218°C hydrothermal fluid (1521 m depth). The temperature of the diffuse fluid was 35°C in 2013.
International District	Diffuse hydrothermal venting emanating from the base and side of the El Guapo metal sulfide hydrothermal edifice emitting 342°C hydrothermal fluid (1515 m depth). The temperature of the diffuse fluid was 24°C in 2013.
Marker N3	Basalt-hosted diffuse hydrothermal vent field flowing from the cooled 2011 eruptive lava flow (1522 m depth). The temperature of the diffuse fluid was 19°C in 2013.
Coquille	Basalt-hosted diffuse hydrothermal venting (1533 m depth) within 10 m of the Vixen anhydrite-forming hydrothermal venting emitting 344°C hydrothermal fluid. The temperature of the diffuse fluid was 34°C in 2013.
North Rift Zone	Basalt-hosted diffuse hydrothermal vent field flowing from the cooled 2015 eruptive lava flow 15 km north of the axial summit caldera (1716 m depth). The temperature of the diffuse fluid was 19°C in 2015.

Supplemental Table 2. Most-probable number (MPN, L^{-1}) estimates of heterotrophs, H_2-producing heterotrophs, methanogens, and non-methanogenic hydrogenotrophs that grow at 55°C and 80°C.

	80°C				55°C			
	2012	2013	2014	2015	2012	2013	2014	2015
**Boca**								
Heterotrophs	690	>33,000	-	-	270	>33,000	-	-
H_2-prod. heterotrophs	ND[a]	ND	-	-	ND	270	-	-
Methanogens	ND	276	-	-	120	276	-	-
Other autotrophs	270	120	-	-	330	630	-	-
Initial total cells ($\times 10^8$, L^{-1})[b]	2.3	5.0	-	-				
**Fuzzy Tubeworm Bush (ASHES)**								
Heterotrophs	2,790	-	-	-	270	-	-	-
H_2-prod. heterotrophs	2,790	-	-	-	270	-	-	-
Methanogens	ND	-	-	-	ND	-	-	-
Other autotrophs	1,290	-	-	-	6,300	-	-	-
Initial total cells ($\times 10^8$, L^{-1})		-	-	-				
**Skadi**								
Heterotrophs	-	3,600	-	-	-	>33,000	-	-
H_2-prod. heterotrophs	-	330	-	-	-	ND	-	-
Methanogens	-	13,800	-	-	-	450	-	-
Other autotrophs	-	1,080	-	-	-	690	-	-
Initial total cells ($\times 10^8$, L^{-1})	-	5.6	-	-				
**International District**								
Heterotrophs	-	>33,000	-	-	-	>33,000	-	-
H_2-prod. heterotrophs	-	120	-	-	-	ND	-	-
Methanogens	-	ND	-	-	-	ND	-	-
Other autotrophs	-	210	-	-	-	4,500	-	-
Initial total cells ($\times 10^8$, L^{-1})	-	0.68	-	-				
**Marker N3**								
Heterotrophs	-	>33,000	-	-	-	>33,000	-	-
H_2-prod. heterotrophs	-	ND	-	-	-	ND	-	-
Methanogens	-	ND	-	-	-	120	-	-
Other autotrophs	-	120	-	-	-	720	-	-
Initial total cells ($\times 10^8$, L^{-1})	-	4.1	-	-				

Table S2 (cont.)

	80°C				55°C			
	2012	2013	2014	2015	2012	2013	2014	2015
**Coquille**								
Heterotrophs	-	6,300	-	-	-	6,300	-	-
H_2-prod. heterotrophs	-	ND	-	-	-	ND	-	-
Methanogens	-	ND	-	-	-	690	-	-
Other autotrophs	-	840	-	-	-	6,300	-	-
Initial total cells ($\times 10^8$, L^{-1})	-	1.4	-	-				
**North Rift Zone**								
Heterotrophs	-	-	-	-	-	-	-	-
H_2-prod. heterotrophs	-	-	-	-	-	-	-	-
Methanogens	-	-	-	2,790	-	-	-	33,000
Other autotrophs	-	-	-	ND	-	-	-	ND
Initial total cells ($\times 10^8$, L^{-1})	-	-	-	3.7				
**CTD cast 25 m over caldera**								
Heterotrophs	-	-	ND	-	-	-	90	-
H_2-prod. heterotrophs	-	-	ND	-	-	-	ND	-
Methanogens	-	-	ND	-	-	-	ND	-
Other autotrophs	-	-	ND	-	-	-	ND	-
Initial total cells ($\times 10^8$, L^{-1})	-	-	0.76	-				
**CTD cast off-summit (1,500 m)***								
Heterotrophs	ND	-	-	-	ND	-	-	-
H_2-prod. heterotrophs	ND	-	-	-	ND	-	-	-
Methanogens	ND	-	-	-	ND	-	-	-
Other autotrophs	ND	-	-	-	ND	-	-	-
Initial total cells ($\times 10^8$, L^{-1})	0.25	-	-	-				

[a]ND, not detected.

[b]Total cell concentration for the hydrothermal fluid sample prior to incubation.

Supplemental Table 3. Growth rate, cell yield based on methane production and cell specific CH_4 production rate for *Methanocaldococcus jannaschii*, *Methanothermococcus thermolithotrophicus*, *Methanocaldococcus bathoardescens* (from Ver Eecke *et al.*, 2013) grown with varying concentrations of NH_4Cl in otherwise nitrogen-free medium. The error represents the 95% confidence interval ($\alpha = 0.05$).

NH_4Cl Concentration	Growth rate (k) (h^{-1})	Yield (Y) (10^{12} cells mol^{-1} CH_4)	CH_4 Prod. Rate (v) (pmol CH_4 $cell^{-1}$ h^{-1})
M. jannaschii:			
47 µM	0.84 ± 0.08	4.14 ± 0.81	0.29 ± 0.06
235 µM	0.79 ± 0.14	3.10 ± 0.44	0.37 ± 0.08
1.8 mM	1.12 ± 0.24	4.04 ± 0.53	0.37 ± 0.09
2.4 mM	0.94 ± 0.16	6.63 ± 1.68	0.20 ± 0.06
4.7 mM	0.64 ± 0.28	4.71 ± 2.15	0.20 ± 0.13
7.0 mM	0.87 ± 0.30	9.21 ± 1.35	0.14 ± 0.05
9.4 mM	0.96 ± 0.34	6.13 ± 1.61	0.23 ± 0.10
M. thermolithotrophicus:			
47 µM	0.75 ± 0.28	4.61 ± 2.10	0.23 ± 0.14
235 µM	0.73 ± 0.18	5.57 ± 2.66	0.19 ± 0.10
2.4 mM	0.64 ± 0.14	1.51 ± 0.43	0.61 ± 0.22
4.7 mM	0.99 ± 0.27	8.79 ± 1.51	0.16 ± 0.05
7.0 mM	0.97 ± 0.24	5.03 ± 2.16	0.28 ± 0.14
9.4 mM	0.78 ± 0.13	3.57 ± 0.36	0.32 ± 0.06
M. bathoardescens:			
24 µM	0	0	0
140 µM	1.53 ± 0.35	1.24 ± 0.38	1.78 ± 0.68
1.8 mM	1.78 ± 0.49	1.56 ± 0.41	1.65 ± 0.63
3.3 mM	1.93 ± 0.86	1.73 ± 0.75	1.61 ± 1.00
4.7 mM	1.72 ± 0.52	1.92 ± 0.74	1.29 ± 0.63
7.0 mM	1.73 ± 0.39	9.26 ± 2.78	0.27 ± 0.10
